# P38 MAPK inhibition prevents polybrene-induced senescence of human mesenchymal stem cells during viral transduction

**DOI:** 10.1371/journal.pone.0209606

**Published:** 2018-12-26

**Authors:** Anastasiia Griukova, Pavel Deryabin, Maria Sirotkina, Alla Shatrova, Nikolay Nikolsky, Aleksandra Borodkina

**Affiliations:** Laboratory of Intracellular Signaling, Institute of Cytology, Russian Academy of Sciences, Saint- Petersburg, Russia; University Medical Center Groningen, NETHERLANDS

## Abstract

The unique capacity of mesenchymal stem cells (MSCs) to migrate to the sites of damage, following intravenous transplantation, along with their proliferation and differentiation abilities make them promising candidates for MSC-based gene therapy. This therapeutic approach requires high efficacy delivery of stable transgenes to ensure their adequate expression in MSCs. One of the methods to deliver transgenes is via the viral transduction of MSCs. However, due to low transduction efficiency of MSCs, various polications are used to promote the association of viral particles with membranes of target cells. Among these polications polybrene is the most widely used one. Unfortunately, viral infection in presence of polybrene was shown to negatively affect proliferation rate of stem cells. The molecular mechanism underlying this effect is not yet uncovered. Therefore, the present study aimed to elucidate the mechanism of this phenomenon as well as to develop an effective approach to overcome the negative impact of polybrene on the properties of human endometrium-derived mesenchymal stem cells (hMESCs) during lentiviral infection. We found that the negative effect on proliferation observed during the viral infection in presence of polybrene is mediated by the polycation itself. Furthermore, we revealed that the treatment with polybrene alone led to the p38 MAPK-dependent premature senescence of hMESCs. These findings allowed us to develop an effective strategy to attenuate the negative polybrene impact on the hMESCs properties during lentiviral infection by inhibiting the activity of p38 MAPK. Importantly, the proposed approach did not attenuate the transduction efficiency of hMESCs, yet prevented polybrene-induced senescence and thereby restored the proliferation of the infected cells. These results provide the plausible means to reduce side effects of polybrene during the viral infection of primary cells, particularly MSCs.

## Introduction

Gene therapy is an actively developing area of modern medicine. It is achieved either by direct transfer of genes into patients or by using living cells as vehicles to deliver genes of interest to sites of injury. In this regard, mesenchymal stem cells (MSCs) are considered to be the most suitable candidates. The integral part of this procedure is an insertion of therapeutic genes into the cells [1]. The common methods to introduce a gene of interest into cells include transfection and viral transduction. While transfection of MSCs fails to be effective, viral transduction is considered as more efficient tool to deliver genetic constructs to MSCs [2, 3].

The disadvantages of the latter approach are the fast loss of the viral bioactivity and their slow diffusion into the host cells. To overcome rapid inactivation of viruses before they reach the target cells, several mechanical approaches have been developed. For example, centrifugation and flow-through transduction were elaborated to elevate the frequency and probability of virus-cell interactions [4]. Despite the high effectiveness of mechanical approaches, the difficulty and costs of such methods limit their application in large-scale investigations or clinical trials [5].

The poor penetration of viruses during infection is largely due to the existence of negative charge on both cell membranes and viral particles [5, 6]. Addition of cationic polymers during viral transduction helps to circumvent this impediment. It is deemed that polycations attenuate the electrostatic repulsion between the cell membrane and virions, so that viruses can more easily adsorb on the cell surface and penetrate the cell [4, 5].

Polybrene (Pb) is the most prevalent among various polycations. Indeed, a wealth of published data demonstrates that the addition of Pb can increase the transduction efficiency several-fold [4–6]. However, there is also information available about the negative influence of Pb on proliferation of different cell lines during viral infection [7, 8]. In accordance with these data, we also have observed the reduced proliferation rates of human endometrium-derived mesenchymal stem cells (hMESCs), the slowdown of their migration as well as the impaired ability to differentiate in osteo- and adipogenic lineages after lentiviral (LV) transduction with the use of Pb [data in print]. However, the underlying mechanism of the Pb impact has not yet been elucidated. Therefore, here we tried to clarify several points: firstly, whether Pb itself might negatively affect hMESCs functioning; secondly, what the molecular cause of its possible impact on hMESCs properties is; thirdly, whether Pb influence would be the same for other primary cells; and, finally, if there is a possibility to overcome the observed negative impact of Pb.

## Materials and methods

### Cell culture

Human mesenchymal stem cells were isolated from desquamated endometrium in menstrual blood from healthy donors (hMESCs, line 2804). The study was reviewed and approved by the Local Bioethics Committee of the Institute of Cytology RAS, protocol #2. The copy of the approval by the Bioethics Committee of the Institute of Cytology is available upon request. hMESCs are characterized by a positive expression of CD 73, CD 90, CD 105, CD 13, CD 29, and CD 44 markers and an absence of expression of the hematopoietic cell surface antigens CD 19, CD 34, CD 45, CD 117, CD 130, and HLADR (class II). Multipotency of isolated hMESCs was confirmed by their ability to differentiate into other mesodermal cell types, such as osteocytes and adipocytes [9]. Human embryonic lung fibroblasts were obtained from the Research Institute of Influenza (St. Petersburg, Russia). Both cell lines were cultured in complete medium DMEM/F12 (Gibco BRL, MD, USA) supplemented with 10% FBS (HyClone, USA), 1% penicillin-streptomycin (Gibco BRL, MD, USA) and 1% glutamax (Gibco BRL, MD, USA) at 37°C in humidified incubator, containing 5% CO_2_. To avoid complications associated with replicative senescence cells at early passages (5–9 for hMESCs and 15–20 for fibroblasts) were used in all experiments.

### Polybrene cell treatment conditions

Polybrene (Pb, hexadiamine bromide) was purchased from Sigma-Aldrich (USA). Pb stock solutions (4 mg/ml) were prepared in water, aliquoted and used for all experiments with no more than three refreeze cycles. For all experiments hMESCs were seeded at 10^5^ in a 35 mm dishes in 1 ml of the complete medium, fibroblasts were seeded at 2.5*10^5^ in the same conditions. Next day after the seeding cells were treated with Pb at indicated concentrations in complete medium for 18 h, after that the medium was replaced with fresh complete medium and cells were maintained for various durations up to analyses.

### HMESCs transduction parameters

Lentiviruses (LV) were produced by co-transfection of HEK293T cells with FgH1tUTG plasmid coding eGFP reporter (a gift from Marco Herold, Addgene plasmid #70183) in combination with the packaging and envelope vectors. For infection hMESCs were seeded and treated as described above with the addition of Pb at concentration 4 mg/ml and viruses at MOI = 5 for 18h. For p38 MAPK inhibition hMESCs were treated with 5 μM SB203580 (Sigma, USA), added to the medium during transduction and to the fresh medium after an infection.

### Flow cytometry analysis

Measurements of cell viability, proliferation, cell size, cell autofluorescence, reactive oxygen species (ROS) and immunophenotyping were carried out by flow cytometry using the CytoFLEX (Beckman Coulter, USA). The obtained data were analyzed using CytExpert software version 1.2. Adherent cells were rinsed twice with PBS and harvested by trypsinization. Detached cells were pooled and resuspended in fresh medium and then counted and analyzed for autofluorescence. In order to assess cell viability, just before analysis 50 μg/ml propidium iodide (PI) was added to each sample and mixed gently. To discriminate live and dead cells, two parameter cytogram (dot plot) was used (PILOG vs FSLOG). The cell size was evaluated by cytometric forward light scattering of PI-negative cells. For surface markers assay cells in an amount of 10^6^ cells per ml were suspended in PBS with 5% fetal bovine serum. Antibodies to CD 90 (#555596, BD Pharmingen, USA), CD 73 (#550257, BD Pharmingen, USA), CD 105 (#560809, BD Pharmingen, USA), CD 44 (#560568, BD Pharmingen, USA), CD 13 (#IM1427U, Beckman Coulter, USA) and CD 146 (#A07483, Beckman Coulter, USA) conjugated with phycoerythrin were used in accordance with manufacturer's recommendations. For the measurement of intracellular ROS levels redoxsensitive probe 2', 7'-dichlorodihydrofluorescein diacetate (H_2_DCF-DA, Invitrogen, CA, USA) was used. Cells were loaded with 10 μM H_2_DCF-DA in serum-free medium and incubated in the dark for 20 min at 37°C, then harvested by tripsinization and suspended in a fresh medium. Cell fluorescence was immediately analyzed by flow cytometry with the peak excitation wavelength for oxidized DCF 488 nm and emission 525 nm. At least 10,000 cells were measured per sample.

### Migration analysis

Cell migration was determined using the xCELLigence RTCA DP system (ACEA Biosciences, USA). Cells were seeded into cell migration plate (CIM-Plate; used with the xCELLigence RTCA DP system) that contains electronically integrated Boyden chambers that provide, in real-time and without the use of labels, quantitative kinetic data for migration. 15*10^3^ hMESCs were seeded in serum-free medium into the upper chamber in 4 days after treatment, the lower chamber was filled with the completed growth medium. Cells moved from the upper chamber towards chemoattractant (serum-containing medium) in the lower chamber, passing through a membrane with 8 μm pores, and then adhered to gold impedance microelectrodes. The resultant change in the impedance signal estimated for 3 days correlated with the number of cells attached to the electrodes.

### SA-β-Gal activity

Cells expressing senescent-associated β-galactosidase were detected by senescence β-galactosidase staining kit (Cell Signaling Technology, USA), according to manufacturer’s instructions. The kit detects β-galactosidase activity at pH 6.0 in cultured cells that is present only in senescent cells and is not found in pre-senescent, quiescent or immortal cells. Quantitative analysis of images was produced with the application of MatLab package, according to the algorithm described in the methodological paper [10]. For each experimental point at least 100 randomly selected cells were analyzed. In 6 days after the treatment cells were harvested by trypsinization and plated at a density of 4.5*10^3^ cells per cm^2^ and additionally cultured for 3 days in order to perform staining in non-confluent cultures.

### Immunoblotting

Immunoblotting analysis was performed as described previously [11]. SDS-PAGE electrophoresis, transfer to nitrocellulose membrane and immunoblotting with ECL (Thermo Scientific, CA, USA) detection were performed according to standard manufacturer’s protocols (Bio-Rad Laboratories, USA). Antibodies against the following proteins were used: glyceraldehyde-3-phosphate dehydrogenase (GAPDH) (clone 14C10) (1:1000, #2118, Cell Signaling, USA), phospho-Rb (Ser807/811) (1:1000, #8516, Cell Signaling, USA), phospho-p53 (Ser15) (clone 16G8) (1:700, #9286, Cell Signaling, USA), p21Waf1/Cip1 (clone 12D1) (1:1000, #2947, Cell Signaling, USA), phospho-ATM (Ser1981) (clone D6H9) (1:1000, #5883, Cell Signaling, USA), phospo-p38 (Thr180/Tyr182) (1:1000, #9211, Cell Signaling, USA) as well as horseradish peroxidase-conjugated goat anti-rabbit IG (1:10000, #7074, GAR-HRP, Cell Signaling, USA) () and antimouse IG (1:10000, #7076, GAM-HRP, Cell Signaling, USA) (). Hyperfilm (CEA) was from Amersham (Sweden). Equal protein loading was confirmed by Ponceau S (Sigma-Aldrich, USA) staining.

### Statistical analysis

Unless otherwise stated, all the quantitative data were shown as M ± SD, the level of statistical significance was set to p < 0.05. To get statistical significance in the difference between two groups two-sided t test or Wilcoxon-Mann-Whitney rank sum test were applied. For multiple comparisons between groups, ANOVA with Tukey HSD were used. All statistical analysis was performed using R software. ***p<0.001, *p<0.05, versus control, §§§<0.001, versus the same type of cells at the other time points or versus (Pb+LV)-treated cells in experiments with SB application.

## Results

### Pb negatively affects hMESCs proliferation, migration and CD146 expression

To assess the effect of Pb on hMESCs, we applied two concentrations of Pb: 4 μg/ml (optimized for LV transduction of hMESCs) and 32 μg/ml (almost the highest concentration tolerated by MSCs based on the search of literature) [12]. The experimental conditions of hMESCs treatment with Pb were the same as for viral infection, except for the absence of LV themselves (for details see the Materials and methods section). Briefly, complete growth medium containing Pb was added to hMESCs for 18 h followed by replacement with fresh medium.

**Fig 1:**
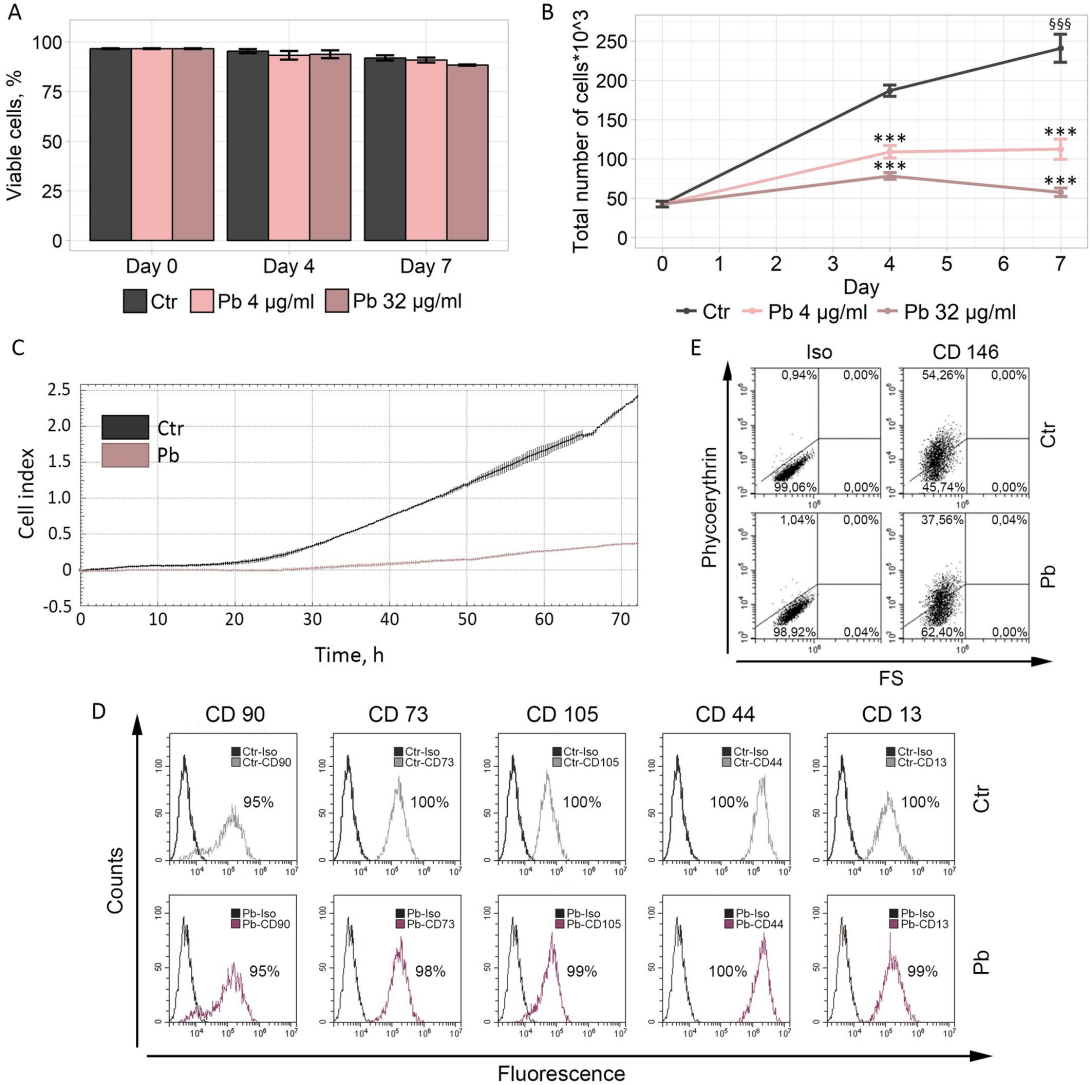
Pb has no effect on hMESCs viability and immunophenotype but has a negative impact on proliferation, migration and CD 146 expression. Except A and B hereinafter “Pb” means the use of Pb in concentration 32 μg/ml. “Ctr”–control cells. (A) Percentage of viable cells evaluated by flow cytometry. (B) Growth curves of Ctr and Pb-treated hMESCs. Cell number was determined by flow cytometry at indicated time points. (C) Migration analysis performed with the use of XCELLigence system. Lines represent cell index M±SD values at multiple time points. (D, E) Expression of common MSCs surface markers in Ctr and Pb-treated cells. “Iso”–isotype control, “FS”–forward scaterring. All results are representatives of at least three independent experiments.

We first estimated cell viability upon Pb treatment using flow cytometry. As shown in Fig 1A, Pb had no substantial impact on hMESCs viability in each concentration tested, as approximately 90% of cells remained PI-impermeable up to 7 days of analysis.

We next assessed whether Pb might affect the proliferation rate of hMESCs. Indeed, we observed a significant reduction in the number of proliferating cells after Pb treatment compared to control hMESCs, as reflected by the growth curves (Fig 1B). Notably, various concentrations of Pb exhibited similar influence on hMESCs proliferation, though the concentration of 32 μg/ml Pb suppressed proliferation more profoundly. Since the concentration of 32 μg/ml Pb did not cause any obvious cytotoxicity, we decided to use this high concentration for subsequent experiments to uncover the molecular cause underlying the anti-proliferative effects of this compound.

To this end, we analyzed the ability of Pb-treated hMESCs to migrate with the use of xCELLigence system. As displayed in Fig 1C, the migration ability of hMESCs after Pb exposure was almost lacking within the 3-day observation period.

Despite of such an obvious and pronounced impact of Pb on hMESCs proliferation and migration, we were not able to reveal any negative effects of such treatment on the expression of main cell surface antigens typical for MSCs, including CD 90, CD 73, CD 105, CD 13 and CD 44 (Fig 1D). However, we detected a significant decrease in the number of CD146-positive cells upon Pb application (Fig 1E). Lately, the reduction of CD146 expression is increasingly regarded as a feature of senescent cells [13].

Collectively, the absence of marked cell death, loss of proliferation, and the decrease in migration together with the attenuation of CD146 expression led us hypothesize that adverse effects of Pb treatment on hMESCs might be mediated by the induction of senescence.

### Pb induces premature senescence in hMESCs

**Fig 2:**
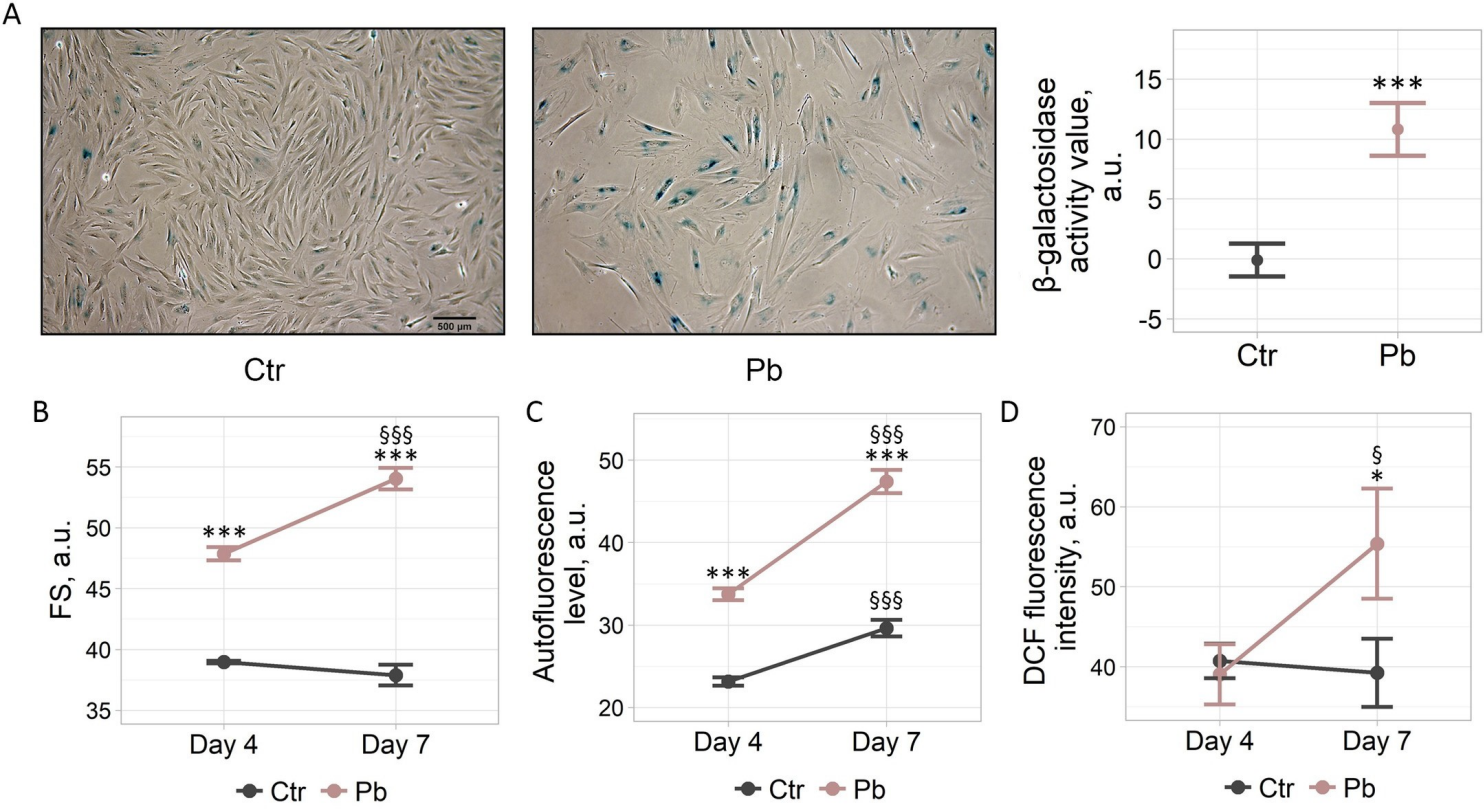
Pb causes the appearance of premature senescence markers in hMESCs. “Ctr”–control cells. “Pb”–Pb-treated cells. (A) SA-β-Gal staining of Ctr and Pb-treated hMESCs. Scale bar is 500 μm and valid for all images. (B, C, D) Cell size, cell autofluorescence and intracellular ROS levels, respectively, determined by flow cytometry. Forward scatter (FS) reflects the average cell size. Intracellular ROS levels were detected after H_2_DCF-DA staining. Representative results of three independent experiments are shown.

In order to study possible contribution of Pb exposure to the induction of senescence, we defined the main features of senescent cells. Firstly, we estimated senescence-associated β-galactosidase (SA-β-Gal) activity in hMESCs upon treatment with Pb. To avoid possible false positive results associated with reaching confluence in culture, both control and Pb-treated hMESCs were re-seeded 6 days after the beginning of the experiment and additionally cultured for 3 days before the staining procedure. We revealed a reliable increase in the number of SA-β-Gal-positive cells within the population of Pb-treated hMESCs, reflecting the augmented activity of this lysosomal enzyme commonly detected in senescent cells (Fig 2A).

Another well established characteristic of senescent cells is hypertrophy. Therefore, we next estimated an impact of Pb on hMESCs size. By analyzing forward scattering of control and Pb-treated cells with the use of flow cytometry we were able to detect almost 1.5-fold increase in cell size upon Pb application (Fig 2B).

Cellular senescence is also known to be accompanied by the impairment of the degradation process and accumulation of fluorescent lipofuscin granules, reflecting damaged macromolecules within the lysosomes [14]. This accumulation might be detected by an increase in autofluoresence intensity. As shown in Fig 2C, in Pb-treated hMESCs autofluorescence increased gradually and was almost twice higher in the 7-day observation period as compared to control cells.

Our previous findings clearly demonstrate that senescence initiation and progression are closely related to the elevated levels of endogenous reactive oxygen species (ROS) [11]. Thus, finally, we assessed the levels of intracellular ROS by H_2_DCF-DA staining. Notably, Pb treatment led to a marked increase in ROS production detected on day 7 (Fig 2D).

Taken together, the obtained data provide a clear confirmation that the reduction in proliferation rates observed during the viral infection is mediated, at least in part, by the progression of Pb-induced senescence.

### Progression of senescence upon Pb treatment is mediated by the activation of p53/p21/Rb pathway

**Fig 3:**
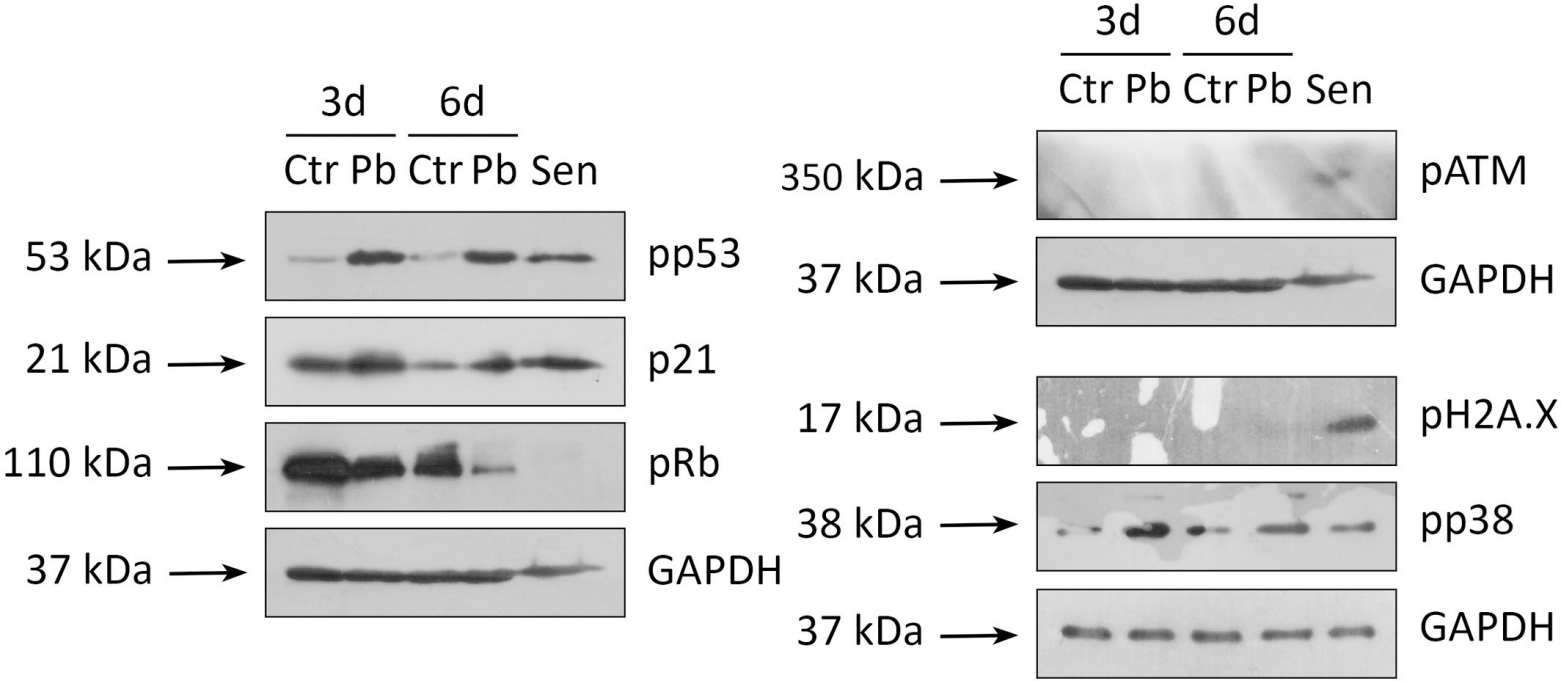
Pb stimulates activation of both p53/p21/Rb and p38 MAPK pathways in hMESCs. “Ctr”–control cells, “Pb”–Pb-treated cells, “Sen”–senescent hMESCs (senescence was induced as described previously [15]). Western blot analysis for pp53, p21, pRb, pp38, pATM and pH2A.X was performed at indicated time points. GAPDH was used as loading control. Representative results of the three experiments are shown in the Figure.

Having established the fact of Pb-dependent induction of senescence, we next investigated the molecular mechanisms underlying this phenomenon. Earlier, we have revealed that permanent cell cycle arrest in prematurely senescent hMESCs under sublethal oxidative stress was mediated by the activation of the p53/p21/Rb pathway [15]. Thus, we checked the phosphorylation/expression status of the indicated proteins upon Pb treatment. As shown in Fig 3, addition of Pb resulted in enhanced phosphorylation of p53, elevated expression of p21 and simultaneous hypophosphorylation of the Rb protein during 6-day observation period.

Previously, we have established that the proliferation arrest of hMESCs during senescence was initiated by the ATM kinase (the main member of the DNA damage response (DDR))-dependent activation of the p53/p21/Rb pathway [11, 16]. In this regard, we examined the phosphorylation status of this kinase in hMESCs upon Pb treatment. Surprisingly, we were not able to detect ATM activation at any time point upon Pb application, suggesting that this was a DDR-independent senescence program (Fig 3). Additionally, we tested the phosphorylation status of another important marker of DDR–H2A.X. As shown in Fig 3, we could not detect phosphorylation of H2A.X upon Pb treatment, which correlates well with lack of ATM activation. According to the literature data, constitutive activation of stress kinase p38 might also induce DDR-independent growth arrest [17]. Therefore, we next checked the activity of p38 MAPK in hMESCs following the Pb treatment. Importantly, we revealed enhanced phosphorylation of p38 MAPK in the Pb-treated cells that persisted at least for 6 days (Fig 3).

The data presented in this part of the Results suggest that, the induction of senescence upon Pb exposure is mediated by the activation of both p38 stress kinase and p53/p21/Rb pathway.

### Pb triggers senescence in human fibroblasts via p53/p21/Rb signaling pathway activation

**Fig 4:**
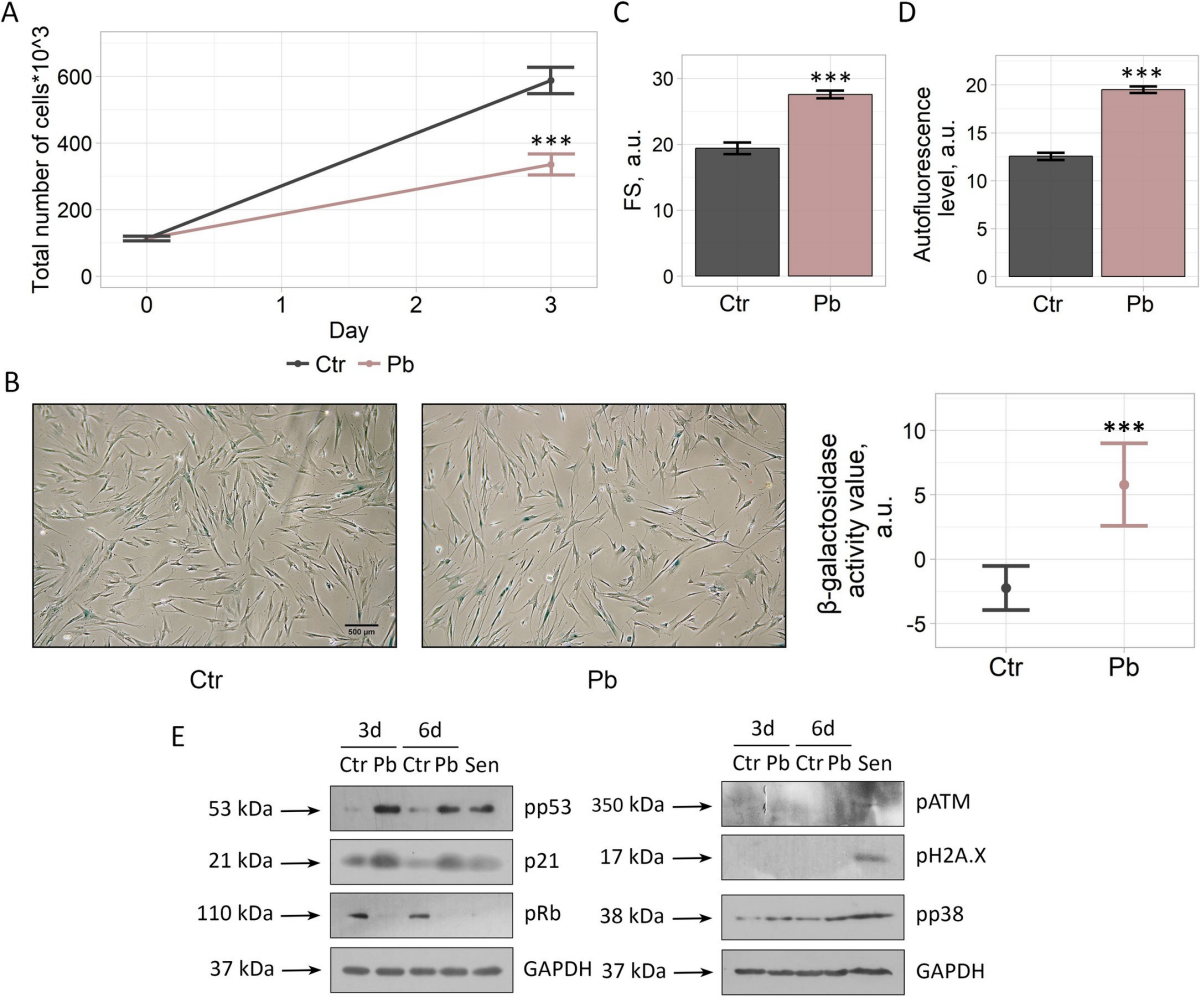
Pb treatment leads to the induction of premature senescence of human embryonic fibroblasts. “Ctr”–control cells. “Pb”–Pb-treated cells. “Sen”–senescent cells. (A) Growth curves, (B) SA-β-Gal staining and quantification, (C) cell size, (D) autofluorescence and (E) western blotting for pp53, p21, pRb, pATM, pH2A.X, and pp38 of Ctr and Pb cells were performed. GAPDH was used as loading control. Scale bar is 500 μm and valid for all images. Representative results of three independent experiments are shown.

In order to verify the results obtained on hMESCs, we checked whether Pb would also induce premature senescence in other type of primary culture cells, namely in human fibroblasts. To this end, we first estimated an impact of Pb on the proliferation of fibroblasts. Indeed, we revealed a significant reduction in the proliferation rate of Pb-treated fibroblasts compared to control cells (Fig 4A). Furthermore, we observed an increase in SA-β-Gal activity, cell size and autofluorescence in fibroblasts upon Pb application (Fig 4B, 4C and 4D). Finally, we observed activation of the canonical p53/p21/Rb pathway in Pb-treated fibroblasts at the indicated time points (Fig 4E). In line with the results obtained for hMESCs, Pb treatment of fibroblasts was not sufficient to induce neither ATM kinase activation nor H2A.X phosphorylation. Moreover, similar to hMESCs, treatment of fibroblasts with Pb caused enhancement of p38 MAPK phosphorylation (Fig 4E). Thus, we concluded that the induction of senescence in the presence of Pb is not a unique reaction of MSCs but is likely a ubiquitous response of primary cells.

### Inhibition of p38 MAP-kinase prevents Pb-induced senescence progression during hMESCs viral transduction

**Fig 5:**
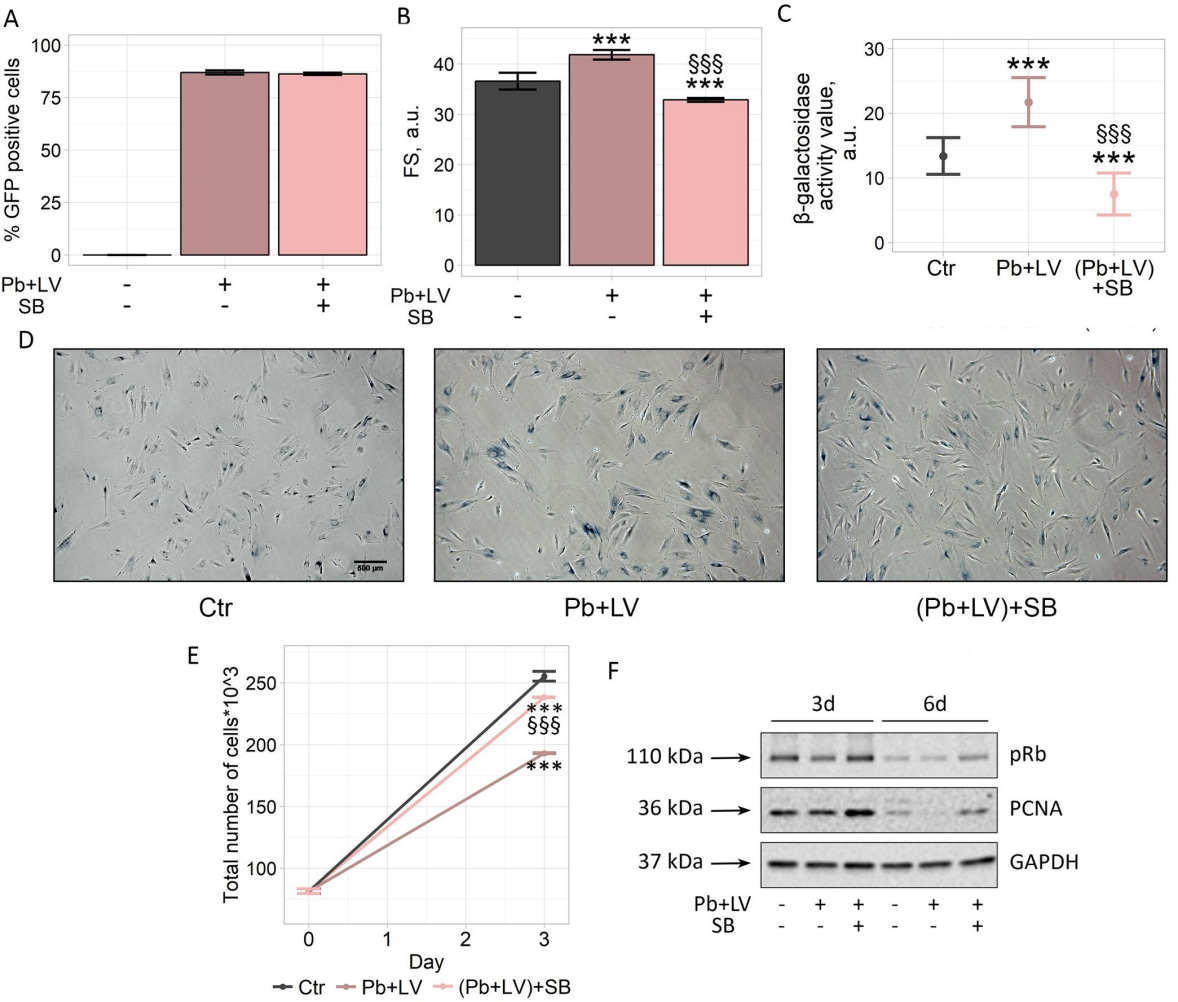
p38 MAPK inhibition reduces negative impact of Pb on the main hMESCs properties during LV transduction. “Ctr”–control cells. “Pb+LV”–the cells infected by LV with 4 μg/ml of Pb. “(Pb+LV)+SB”–the cells infected by LV with 4 μg/ml of Pb additionally supplied with 5 μM SB. (A). The effectiveness of hMESCs transduction estimated by FACS analysis of the green fluorescence intensity. (B) Cell size. (C, D) SA-β-Gal quantification and staining. Scale bar is 500 μm and valid for all images. (E) Growth curves. (F) Rb phosphorylation and PCNA protein expression at indicated time points. GAPDH was used as loading control. All results are representatives of at least three independent experiments.

The results presented above clearly demonstrate that reduction in the proliferation rate observed during LV infection of hMESCs is mediated primarily by the Pb-induced senescence. In order to overcome this negative effect accompanying viral infection, we sought for the way to prevent senescence. As described above, progression of senescence in Pb-treated hMESCs is accompanied by the persistent upregulation of p38 MAPK phosphorylation. In our previous findings we have revealed that p38 MAPK inhibition partially prevents oxidative stress-induced senescence of hMESCs [11]. Therefore, we decided to test whether the treatment of cells with SB203580 (SB), the selective inhibitor of p38 kinase activity, would be sufficient to reduce Pb-induced senescence during LV infection of hMESCs. To this end, we performed a set of experiments of LV transduction in presence of 5 μM SB (concentration chosen previously and sufficient to prevent H_2_O_2_-induced senescence of hMESCs), according to the procedure described in Materials and methods section. We first checked whether SB application would affect transduction efficiency. There was no impact of SB treatment on the GFP fluorescence, reflecting infection efficiency, at least during the 6-day observation period (Fig 5A). We then estimated the main features typical for senescent cells. As shown in Fig 5, SB application resulted in cell size decrease (Fig 5B), reduction in SA-β-Gal activity (Fig 5C and 5D) and, finally, in partial restoration of proliferation of hMESCs infected with Pb+LV (Fig 5E). In line with the proliferation recovery, we detected an increase in Rb phosphorylation and enhancement in the expression of proliferation antigen (PCNA) in SB-treated cells compared to non-treated ones during viral transduction (Fig 5F). Notably, SB had the same effects on Pb-treated cells in the absence of LV as well (S1 Fig). Summarizing the described data, we can conclude that SB application during LV infection of MSCs might be a good strategy to attenuate the negative Pb impact. On the one hand, it partially reverts Pb-induced senescence, and on the other hand, it does not affect the transduction efficiency.

## Discussion

Previously, we have shown that the presence of Pb significantly increases the transduction efficiency of hMESCs [data in print]. However, we and others have observed that the presence of Pb during viral infection adversely affects the main stem cell properties [7, 8, 18]. Interestingly, when another polycation, protamine sulfate, was used for transduction we did not detect any negative effects on the hMESCs properties. This led us to hyphothesize that the observed negative influence is rather mediated by Pb than by other transduction parameters. Despite several reports showing the negatory impact of Pb on proliferation of MSCs during viral infection, the underlying molecular mechanisms of this phenomenon remained uncovered [8, 19, 20]. Therefore, in the present study we focused on the investigation of the molecular causes of Pb effects on the fate of hMESCs and on possible ways to reduce it.

We revealed that cell treatment with Pb alone is sufficient to induce reduction of the proliferation rate. There are at least two common causes of proliferation loss, namely cell death and irreversible cell cycle block. The choice between these cellular programs depends largely on the cell type [21, 22]. For example, epithelial cells are more prone to apoptosis than to triggering irreversible cell cycle block [23]. On the contrary, stromal cells are resistant to apoptosis and are more likely to enter the state of irreversible cell cycle block [22]. Thus, it was logical to assume that the negative Pb impact might be also cell type dependent. Indeed, Pb reduced proliferation of rat cochlear hair cells [19] and human tracheal epithelial cells due to cell death induction [20]. However, Pb treatment of bone marrow MSCs and human epidermal keratinocyte stem/progenitor cells led to proliferation loss with no detectable cell death, which argues in favor of cell cycle arrest. Moreover, according to Lin’s data, MSCs were not able to regain proliferative capabilities even 3 weeks after Pb treatment, suggesting the irreversibility of cycle block induced by Pb [8]. In case of Pb-treated hMESCs we did not reveal any cell death, indicating that the proliferation arrest observed is likely mediated by cell cycle block.

These results led us to suspect that Pb might induce senescence. In line with this hypothesis, we observed the impairment of proliferation and migration, enhanced SA-β-Gal activity, cell hypertrophy, elevated autofluorescence and enhanced endogeneous ROS levels. These features correlated with activation of the p53/p21/Rb pathway in hMESCs upon Pb treatment. According to the literature data, our results reflect all the main features typical for senescent cells [11, 15, 16, 21, 24, 25, 26]. Thus, the underlying cause of Pb negative effects on hMESCs properties is the initiation of premature senescence. In order to verify the hypothesis, we checked Pb effects on the other type of stromal cells–fibroblasts. Indeed, upon Pb treatment of fibroblasts we observed a reduction of proliferation along with all other senescence markers. As in hMESCs, upon Pb exposure of fibroblasts we detected activation of the p53/p21/Rb pathway that mediated cell cycle block. These data clearly shows that the Pb application leads to premature senescence in different primary cell cultures, namely in hMESCs and in fibroblasts.

Further, we focused on the molecular causes of senescence induced by Pb treatment. The most frequent mechanism triggering senescence is exerted via p53/p21/Rb pathway associated with DDR activation [11, 25]. Surprisingly, we did not observe phosphorylation of the major players of DDR, ATM kinase and H2A.X, neither in Pb-treated hMESCs nor in fibroblasts. These results suggest that p53/p21/Rb-mediated cell cycle arrest of both cell types upon Pb exposure might be DDR-independent. However, we detected an activation of p38 MAPK upon Pb treatment. One possible explanation is that, being a polycationic polymer, Pb may alter the cell surface charge and disrupt the transmembrane potential, preventing the influx of cations, hence leading to variations in extracellular osmolarity [4]. P38 MAPK is known to be specifically regulated by changes in environmental osmolarity, thus playing an important role in the cellular response to osmotic alterations [27–29]. At the same time, p38 stress kinase might be involved in the establishment of senescence due to its ability to activate the p53 growth arrest pathway [17]. Moreover, constitutive p38 MAPK activity was shown to be sufficient to induce DDR-independent growth arrest [17, 30].

It should be emphasized that the present study is the first attempt to elucidate molecular reasons and the underpinning mechanisms of Pb negative impact on cellular proliferation. The discovery of this molecular mechanism allowed us to develop the effective approach to overcome Pb side effects during hMESCs transduction. We speculated that p38 inhibition might prevent hMESCs senescence and thus attenuate the adverse Pb influence during LV infection. In fact, when p38 activity was inhibited, we observed reduction of the main senescence markers and, finally, we achieved the most desired result–proliferation restoration of hMESCs infected in presence of Pb. Noteworthy, applied p38 inhibitor had no impact on the efficacy of LV transduction. It should be noted that in the previous studies revealing the negative impact of Pb on cellular proliferation authors also tried to solve this issue [11, 18]. For example, Lin et al., tried to culture MSCs in the presence of a potent mitogen FGF-2 [11]. However, FGF-2 did not abrogate negative effect, which is in line with our finding that Pb induces premature senescence in the primary cells. Senescence is defined as the state of fully irreversible growth arrest that can not be prevented even by the additions of mitogens [26]. Thus, in the presence of FGF-2 MSCs were not able to regain proliferation. Another attempt to restore proliferation was performed on keratinocyte stem/progenitor cells by Nanba et al. [18]. The authors were able to recover proliferation of keratinocytes infected in the presence of Pb via inhibition of Rho-associated kinase that is essential for cell cycle progression and senescence. However, the application of ROCK inhibitor interfered with the lentiviral transduction.

Summarizing the results obtained in this study, we can conclude that the negative effects of Pb on hMESCs properties are mediated by the induction of p38 MAPK-dependent premature senescence. Hence, p38 MAPK inhibiton is sufficient to prevent the Pb-induced proliferation loss during LV transduction of hMESCs.

## Supporting information

S1 Fig p38 MAPK inhibition reduces negative impact of Pb on the main hMESCs properties in the absence and presence of LV. “Ctr”–control cells. “Pb”–Pb-treated cells, “SB”–SB-treated cells. The remaining abbreviations are the same as in the previous figure legends. (A) Cell size and (B) growth curves. (C) Rb phosphorylation and PCNA protein expression levels at indicated time points. GAPDH was used as loading control. (D) SA-β-Gal staining and its quantification. Scale bar is 500 μm and valid for all images. All results are representatives of at least three independent experiments.
(TIF)
