# Supplementary figures and images for: P38 MAPK inhibition prevents polybrene-induced senescence of human mesenchymal stem cells during viral transduction
Source: PLoS One. 2018 Dec 26;13(12):e0209606. doi: 10.1371/journal.pone.0209606 (PMC6306270; doi:10.1371/journal.pone.0209606)

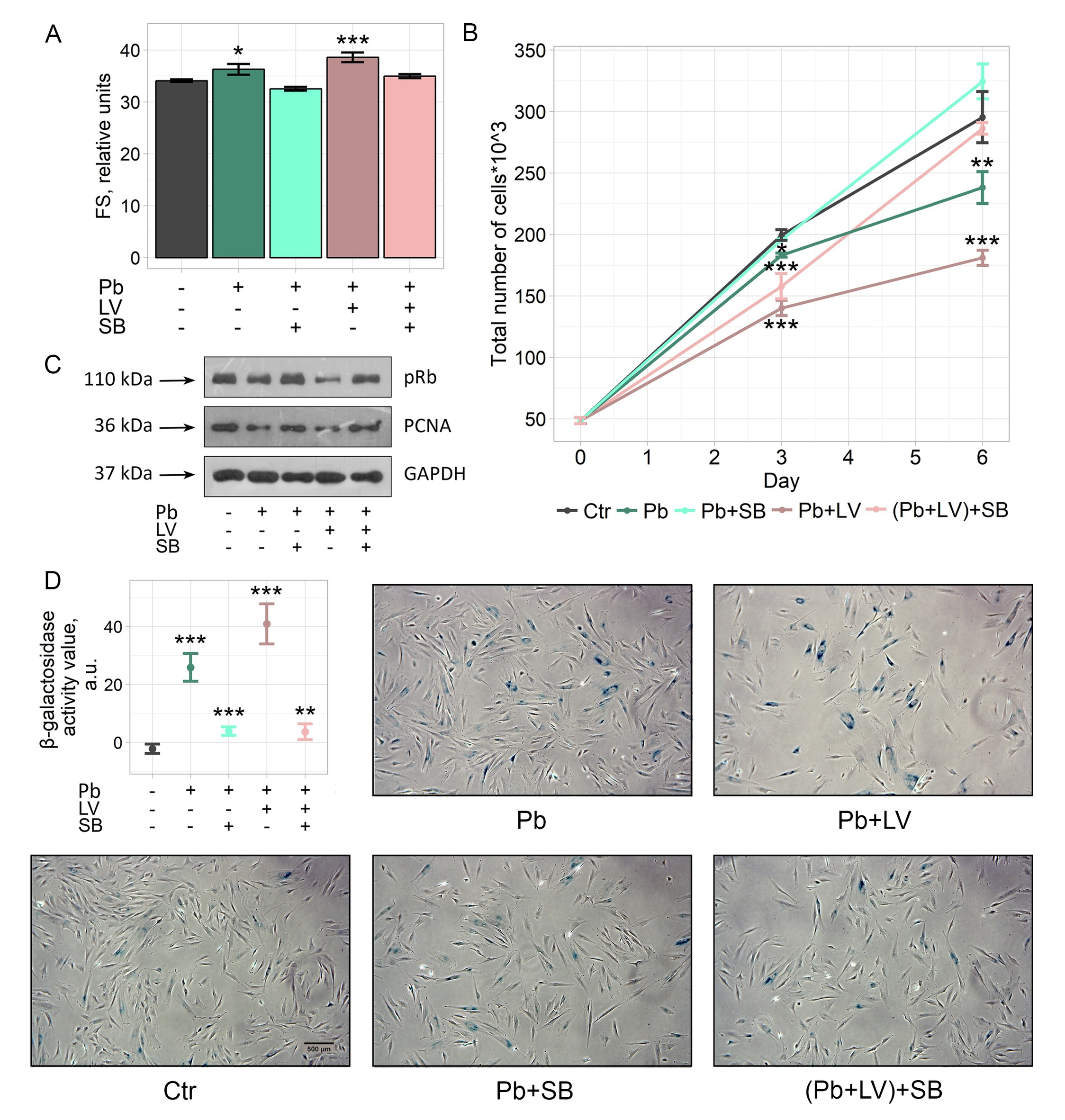

Supplement: S1 Fig — “Ctr”–control cells. “Pb”–Pb-treated cells, “SB”–SB-treated cells. The remaining abbreviations are the same as in the previous figure legends. (A) Cell size and (B) growth curves. (C) Rb phosphorylation and PCNA protein expression levels at indicated time points. GAPDH was used as loading control. (D) SA-β-Gal staining and its quantification. Scale bar is 500 μm and valid for all images. All results are representatives of at least three independent experiments. (TIF) [file pone.0209606.s001.tif]
